# *Aedes albopictus* Mosquitoes, Yucatan Peninsula, Mexico

**DOI:** 10.3201/eid1803.111626

**Published:** 2012-03

**Authors:** Jaime Salomón-Grajales, Gerardo V. Lugo-Moguel, Víctor R. Tinal-Gordillo, Jorge de La Cruz-Velázquez, Barry J. Beaty, Lars Eisen, Saul Lozano-Fuentes, Chester G. Moore, Julián E. García-Rejón

**Affiliations:** Servicios Estatales de Salud, Quintana Roo, Mexico (J. Salomón-Grajales);; Departamento de Vectores de la Jurisdicción Sanitaria No 2, Quintana Roo (G.V. Lugo-Moguel, V.R. Tinal-Gordillo, J. de La Cruz-Velázquez);; Colorado State University, Fort Collins, Colorado, USA (B.J. Beaty, L. Eisen, S. Lozano-Fuentes, C.G. Moore);; Universidad Autónoma de Yucatan, Yucatan, Mexico (J.E. García-Rejón)

**Keywords:** Aedes albopictus, Aedes aegypti, mosquitoes, dengue virus, viruses, Yucatan Peninsula, Mexico

**To the Editor:** We collected Asian tiger mosquitoes, *Aedes albopictus* (Skuse), in Cancun in the Yucatan Peninsula of Mexico in September 2011. This mosquito is a nuisance biter of humans and a vector of numerous arboviruses, including those causing dengue, yellow fever, and chikungunya (*1*).

*Ae. albopictus* mosquitoes, which are native to Southeast Asia, emerged in the continental United States in 1985 and thereafter spread rapidly across the southeastern United States and into northern Mexico (*2*,*3*). These mosquitoes have also been found in the states of Tamaulipas, Coahuila, and Nuevo Leon in northern Mexico, Chiapas in southern Mexico, and south of Mexico in Guatemala and Belize (*3*–*9*). These findings are now complemented by our collection of *Ae. albopictus* mosquitoes from Cancun in Quintana Roo State, which with Yucatan and Campeche States compose the Yucatan Peninsula. A previous study of the mosquito fauna of Quintana Roo conducted in 2006 did not report any *Ae. albopictus* mosquitoes (*10*).

During September 2011, *Ae. albopictus* mosquitoes were collected from a cemetery in Cancun, which is located in the eastern part of the Yucatan Peninsula (21°8.53′Ν, 86°52.79′W) (Figure). The collection location was shaded by trees. Water in containers from which larvae were collected had an average temperature of 24.5°C and a pH of 8.5. The larval collection included ≈30 specimens of different developmental stages that were collected from vases and other artificial containers in the cemetery. The containers were examined as part of routine surveillance activities by Servicios Estatales de Salud de Quintana Roo. Larvae suspected to be those of *Ae. albopictus* mosquitoes were reared to adults for identification, and a colony of *Ae. albopictus* mosquitoes from Cancun was established.

**Figure:**
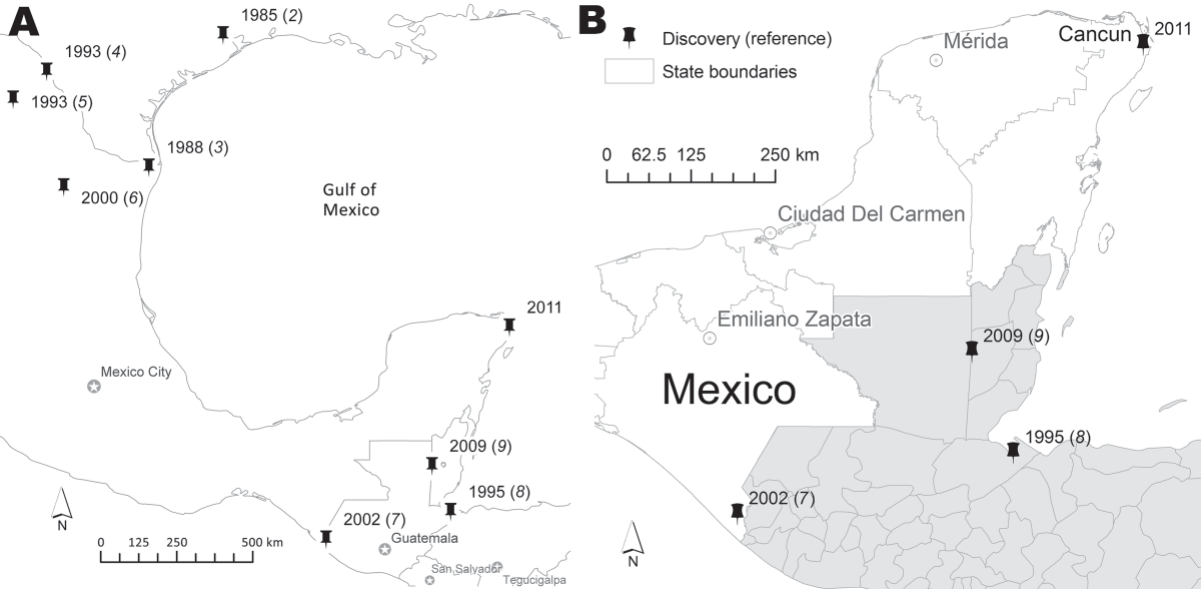
Notable locations (pushpins) in Mexico, the United States, and Central America where *Aedes albopictus* mosquitoes were collected and year of the first collection (reference) (A), including the current collection in 2011 from Cancun, Quintana Roo State, Mexico (B). Shaded areas indicate countries in Central America (Guatemala, Belize, Honduras, and El Salvador).

F_0_ or F_1_ adult specimens were confirmed to be *Ae. albopictus* mosquitoes by species identification at Servicios Estatales de Salud de Quintana Roo (Quintana Roo, Mexico), Universidad Autónoma de Yucatan (Merida, Mexico), and Colorado State University (Fort Collins, CO, USA). The initial mosquito larval collection was composed of 26 *Ae. albopictus*, 3 *Ae. aegypti*, and 1 *Culex* sp. In addition, 6 *Ae. albopictus* female mosquitoes were collected from the cemetery by landing catches.

Finding *Ae. albopictus* mosquitoes in Cancun was not surprising because these mosquitoes have been found in nearby Belize (*9*). Cancun is also a major port for ships carrying tourists and goods that originate in areas to which *Ae. albopictus* mosquitoes are endemic, including Florida and Texas. Nevertheless, the introduction of *Ae. albopictus* mosquitoes into Cancun and the high potential for establishment and spread across the Yucatan Peninsula has major public health implications.

The Yucatan Peninsula is hyperendemic for dengue, with all 4 dengue virus (DENV) serotypes circulating in this region. Should *Ae. albopictus* mosquitoes persist in this region, they may spread and come to play a secondary role to *Ae. aegypti* mosquitoes as local vectors of DENV. *Ae. albopictus* mosquitoes may also change local virus transmission dynamics. For example, DENV transmission may be intensified in rural areas because *Ae. albopictus* mosquitoes are more likely than *Ae. aegypti* mosquitoes to be found in this setting. *Ae. albopictus* and *Ae. aegypti* mosquitoes also may differ in their potential for vertical transmission of DENV, which could affect virus transmission dynamics, especially during interepidemic periods or parts of the year that have low mosquito activity and infrequent human–mosquito contact. Other concerns regarding introduction of *Ae. albopictus* mosquitoes into the Yucatan Peninsula are their role as an aggressive nuisance biter of humans, which may necessitate intensified mosquito control to protect the local tourist industry; and their potential role as a vector of chikungunya virus, which is a major threat to immunologically naive populations in the Americas should the virus emerge there.

Introduction of *Ae. albopictus* mosquitoes into the Yucatan Peninsula requires research on local biology of the mosquito and their potential role as an arbovirus vector in this part of Mexico. Studies are needed to determine how fine-scale spatial segregation of *Ae. albopictus* and *Ae. aegypti* mosquitoes might result from competition for containers that serve as larval development sites, from differential survival related to container type, and from hydrologic microclimates or nutrient conditions. One possible scenario is for *Ae. albopictus* mosquitoes to outcompete and exclude *Ae. aegypti* mosquitoes from certain settings. Other issues include how effectively *Ae. albopictus* mosquitoes can transmit locally circulating DENV strains and, because this species bridges the transitional zone from urban to forested environments and may bite a wide range of mammals, what role it might play in the urban emergence of arboviruses that are currently restricted to sylvatic forest transmission cycles in the Yucatan Peninsula.
